# The possible role of sodium leakage channel localization factor-1 in the pathophysiology and severity of autism spectrum disorders

**DOI:** 10.1038/s41598-023-36953-0

**Published:** 2023-06-16

**Authors:** Sarah Al-Mazidi, Laila Al-Ayadhi, Fatmah Alqahtany, Amani Abualnaja, Abdullah Alzarroug, Turki Alharbi, Karim Farhat, Ahmad AlMnaizel, Afaf El-Ansary

**Affiliations:** 1 Physiology Department, College of Medicine, Imam Mohammad Ibn Saud Islamic University, P.O.Box: 5701, Riyadh, 11432 Saudi Arabia; 2 Physiology, King Saud University College of Medicine, Riyadh, Saudi Arabia; 3 Autism Research and Treatment Center, King Saud University College of Medicine, Riyadh, Saudi Arabia; 4 Hematopathology Unit, Department of Pathology, College of Medicine, King Saud University, King Saud University Medical City, Riyadh, Saudi Arabia; 5 College of Medicine, Imam Muhammad bin Saud Islamic University, Riyadh, Saudi Arabia; 6 Cancer Research Chair, College of Medicine, King Saud University, Riyadh, Saudi Arabia; 7 Research office, John Hopkins Aramco Healthcare, Dahran, Saudi Arabia

**Keywords:** Physiology, Biomarkers, Molecular medicine

## Abstract

Autism spectrum disorder (ASD) is a neurodevelopmental disorder characterized by social, stereotypical, and repetitive behaviors. Neural dysregulation was proposed as an etiological factor in ASD. The sodium leakage channel (NCA), regulated by NLF-1 (NCA localization factor-1), has a major role in maintaining the physiological excitatory function of neurons. We aimed to examine the level of NLF-1 in ASD children and correlate it with the severity of the disease. We examined the plasma levels of NLF-1 in 80 ASD and neurotypical children using ELISA. The diagnosis and severity of ASD were based on the Diagnostic and Statistical Manual of Mental Disorders, Fourth Edition (DSM-IV), Childhood Autism Rating Score, Social Responsiveness Scale, and Short Sensory Profile. Then, we compared the levels of NLF-1 with the severity of the disease and behavioral and sensory symptoms. Our results showed a significant decrease in the plasma levels of NLF-1 in ASD children compared to neurotypical children (*p* < 0.001). Additionally, NLF-1 was significantly correlated with the severity of the behavioral symptoms of ASD (*p* < 0.05). The low levels of NLF-1 in ASD children potentially affect the severity of their behavioral symptoms by reducing neuron excitability through NCA. These novel findings open a new venue for pharmacological and possible genetic research involving NCA in ASD children.

## Introduction

Autism spectrum disorder (ASD) is a construct used to describe a neurodevelopmental disorder with a specific combination of impaired social communication and repetitive, stereotypical behavior. Multifactorial etiopathogenesis was linked to ASD, including genetic, immune, and environmental factors. Other factors related to ASD are neurogenic dysregulation observed in neuroimaging and electrophysiological studies^1^. Neurogenic dysregulations were also reported by studying specific neurogenic biomarkers that detect neuroinflammation, abnormal neural and synaptic excitation and channelopathy^2,3^.

Neural excitation, which is affected in ASD, might be caused by channelopathy. The sodium leakage channel (NCA) is one of the channels that affect neural function. It is highly expressed in the central nervous system and associated with synapse development. The NCA ion channel interacts with the M3 muscarinic receptor upon activation by acetylcholine. A protein identified to regulate neural membrane potential and excitability through NCA is known as protein NLF-1 (NCA localization factor-1). NLF-1 is an endoplasmic reticulum-resident protein that interacts with NCA, affecting its folding^4^. Decreased NLF-1 or NCA induces hyperpolarization and reduced neural excitability^5^.

Several neurodegenerative and psychiatric disorders were found to be associated with NCA dysfunction through NLF-1, such as attention-deficit/hyperactivity disorder (AD/HD) and depression^4^. Similar symptoms in AD/HD and depression were reported in ASD children^6,7^. Additionally, working or short-term memory, which is affected in ASD children, requires a normally functioning NCA^8,9^. In addition, studies have reported that abnormal NCA function leads to speech impairment and cognitive delay, which are early signs of ASD^10,11^.

This is the first study to investigate the effect of NLF-1 in ASD children. Given the association of neural dysfunction in ASD patients and the severity of the disease, we aimed to investigate the correlation between ASD and NLF-1 as a disorder biomarker and determine its correlation with the severity of the symptoms of ASD.

## Methods

This study was conducted with the approval of the Institutional Review Board of the Faculty of Medicine, King Saud University, according to the most recent Declaration of Helsinki. The parents of all participants gave informed consent before the assessment.

### Participants

A total of 80 children were recruited into two groups, the ASD group and the neurotypical group. The ASD group included 40 male ASD children aged from 3 to 12 years old recruited by the Autism Research and Treatment Center, Faculty of Medicine, King Saud University and King Khalid University Hospital. Age- and sex-matched neurotypical children were recruited from the pediatrics clinic of King Khalid University Hospital for routine follow-up.

This study included children with confirmed ASD diagnoses based on the Diagnostic and Statistical Manual of Mental Disorders, Fourth Edition (DSM-IV). Any child with an active illness, infectious disease or neuropsychiatric disorder was excluded from the study.

### Assessing autistic symptoms and severity

#### Childhood Autism Rating Score (CARS)

The Childhood Autism Rating Score is a widely used observational rating scale for the diagnosis and detection of autism and to discriminate children with ASD from other types of intellectual delays^12^. The CARS consist of 15 domains assessing behaviors associated with autism. Each domain is scored on a scale from 1 (normal) to 4 (severe abnormality). Children with a total score of 30 a below indicate that the child is not autistic.

A score between 30 and 36.5 indicated mild to moderate autism. Severe autism is indicated with a CARS score between 37 and 60. The CARS domains are categorized into items relating to people, emotional response, imitation, body use, object use, listening response, fear or nervousness, verbal communication, nonverbal communication, activity level, level and consistency of intellectual response, adaptation to change, visual response, taste, smell and touch response, and general impressions.

#### Social Responsiveness Scale (SRS)

The Social Responsiveness Scale is a valid scale to measure the severity of ASD symptoms and to distinguish ASD behavior from other disorders accompanied by disruptive behavior disorders. The scale comprises 65 items that parents or teachers can complete within 15–20 min^13^. It is a quantitative questionnaire completed by the parents or the teacher based on the child's observed behavior for the past six months.

The scale assessment areas include autistic mannerisms, social awareness, cognition, communication, and motivation. It has an ordinary 4-point scale of "0" (not true) to "3" (almost always true). Then, the scores are categorized to a cutoff score of 60 as follows: a mild to moderate range of social impairment is for children scored 60–75, and severe social impairment is for children scored 76 or higher.

#### Short sensory profile (SSP)

The short sensory profile questionnaire contains 38 items categorized into seven categories. Each category can classify a certain level of sensory abnormality measured on a 5-point Likert scale (1 being "Always" and five being "Never") ^14^. The subscales are in the following areas: tactile sensitivity (7 items), taste/smell sensitivity (4 items), movement sensitivity (3 items), seeking sensation (7 items), auditory filtering (6 items), low-energy levels (6 items), and visual/auditory sensitivity (5 items). The SSP final score classifies children with normal sensory responses and children with sensory disabilities of different levels. Lower scores indicate a more sensory disability. Children who scored lower than 142 were children with a severe sensory disability, those who scored between 143 and 152 were children with mild to a moderate sensory disability, and those who scored above 153 were children with typical sensory performance.

### Measurement of plasma NLF-1

Blood was collected in EDTA tubes from participants in both the ASD and control groups. Then, the samples were centrifuged to isolate the plasma, aliquoted, and stored at − 80 °C until analysis. The plasma concentration of NLF-1 was measured by ELISA using a commercial kit according to the manufacturer's instructions (human NLF-1 ELISA kit, Wuhan EIAab Science Company, Wuhan, China). Samples and standards were added to the precoated plate with antibodies specific for NLF-1 and analyzed in duplicate for each protein of interest.

### Statistical analysis

Data are presented as the mean ± standard deviation. The Mann–Whitney test was used for non-normally distributed data to compare the plasma levels of NLF-1 in two groups, ASD and control. Also, Spearman correlation was used when comparing two variables, for example, age with NLF-1. Statistical analysis was performed with SPSS software, version 25. Two-tailed tests for statistical significance were performed where p < 0.05 was considered significant. Statistical significance was denoted as *p < 0.05; **p < 0.01.

### Ethical approval

Institutional Review Board and Guidelines of Health Sciences Colleges Research on Human Subjects, King Saud University, College of Medicine. Ref. No. 22/0122/IRB approved this study. An informed consent form was obtained from all participant's parents or legal guardians prior to participation for approving processing and publishing data.

## Results

The mean age of the ASD group was 6.3 ± 2 years, and the mean age of the control group was 5.8 ± 3 years. Other demographics, including a family history of psychiatric and autoimmune disorders, are shown in Table 1. The ASD children were subcategorized according to the severity of the disease (CARS), behavioral symptoms (SRS), and sensory disability (SSP) into mild, moderate, and severe categories. The mean CARS scores for the mild to moderate ASD group were 32.5 ± 1.7 and 42.6 ± 6.4, respectively. The mean SRS score was 65.9 ± 11 in the mild to moderate group and 130.9 ± 3 in the severe group. The SSP scores were 168.5 ± 3 for the typical (normal sensory performance) ASD group and 122.9 ± 22 for the abnormal ASD group.

**Table 1:** Study demographics.

	Control (mean ± SD)	ASD (mean ± SD)
Age (years)	5.8 ± 3	6.3 ± 2
Family medical history	% (n)	% (n)
Depression	7 (18)	–
Bipolar	5 (12.5)	–
Schizophrenia	3 (7.5)	–
Rheumatoid arthritis	4 (10)	2 (5)
Systemic lupus erythematosus	3 (7.5)	1 (0.4)
Multiple sclerosis	2 (5)	2 (5)

**Figure 1:**
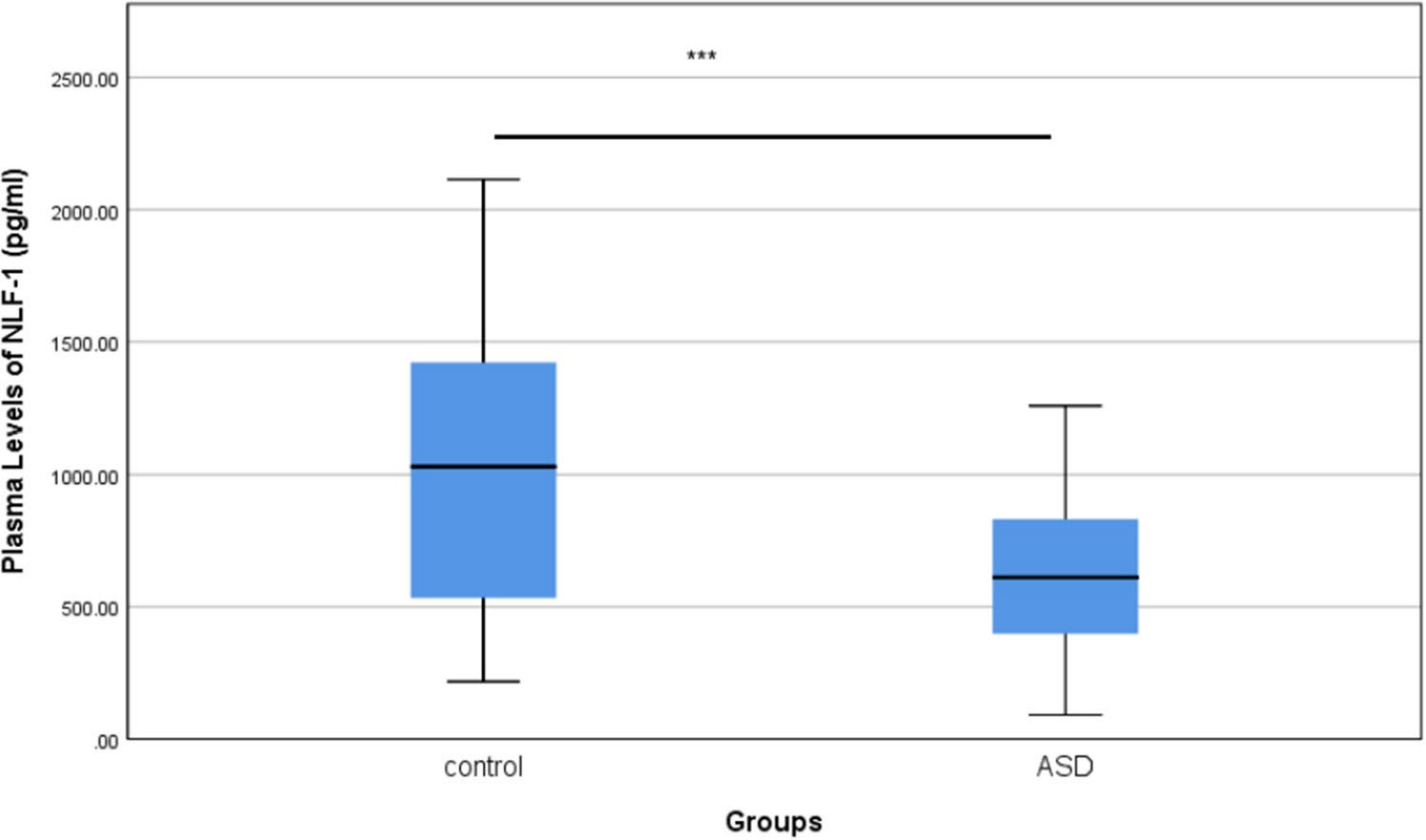
The comparison between plasma levels of NLF-1 in ASD group and neurotypical group.

Table 2 shows the plasma levels of NLF-1 in the ASD and control groups. Figure 1 demonstrates a significant correlation between the plasma levels of NLF-1 in the ASD and control groups (p < 0.001). Our results showed that plasma levels of NLF-1 are significantly decreased in ASD children compared to neurotypical children. The mean difference between the two groups was 387.1 pg/ml.

**Table 2:** Plasma levels of NLF-1 in ASD and neurotypical groups.

Biomarker	Control (mean ± SD)	ASD (mean ± SD)	*p* value
NLF-1 (pg/ml)	1041.4 ± 543.2	654.4 ± 320.4	< 0.001

**Figure 2:**
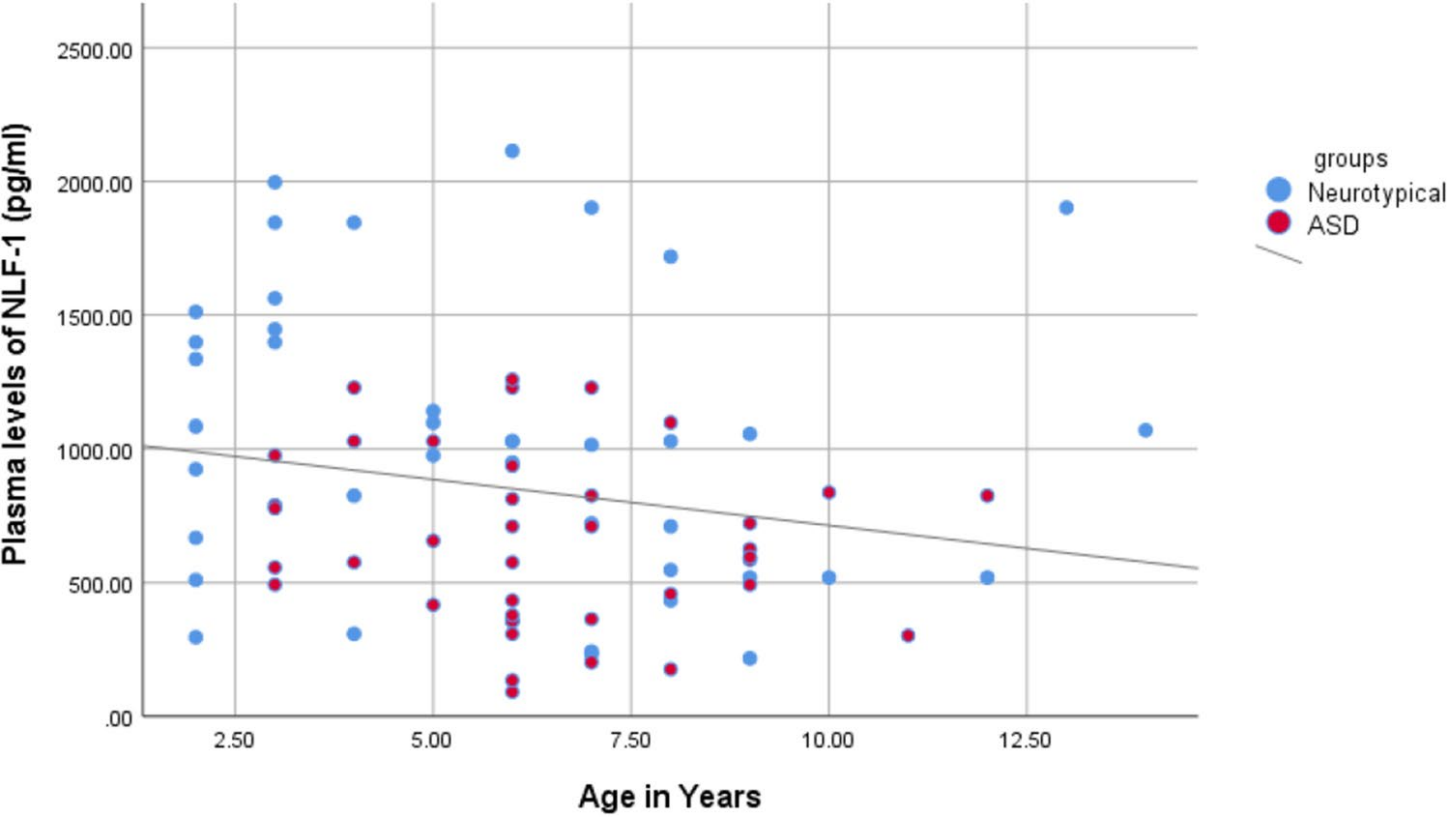
The correlation between plasma levels of NLF-1 and age in both ASD group and neurotypical group.

Figure 2 shows a significant negative correlation. Even though the data are not normally distributed, correlation analysis showed a negative correlation between age and NLF-1 in both groups (p < 0.05), r = − 0.250.

**Figure 3:**
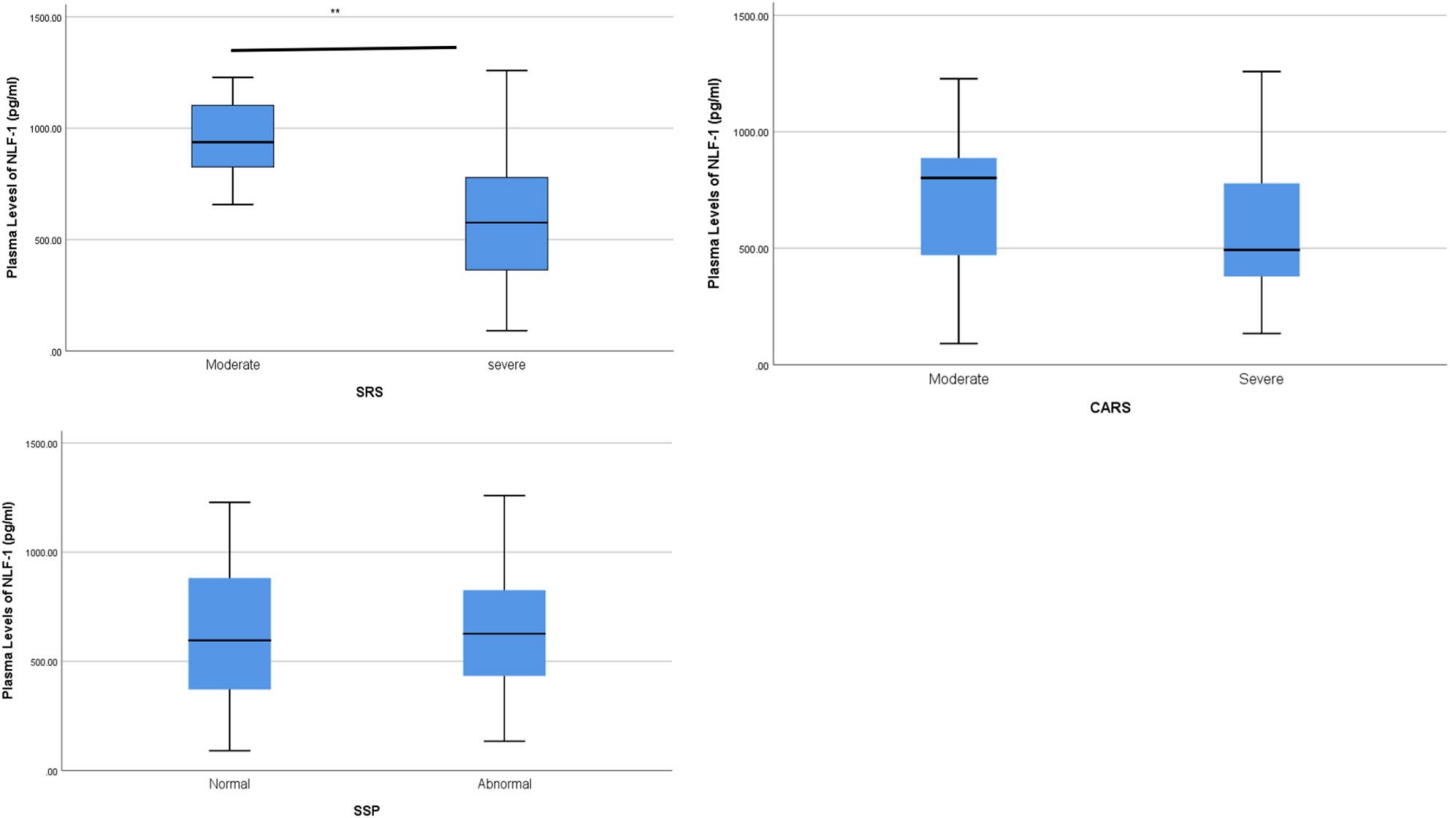
Plasma levels of NLF-1 in ASD subgroups of CARS, SRS and SSP.

Figure 3 shows the subcategories of ASD according to SSR, CARS and SSP scores and Table 3 shows the correlation between ASD subgroups with plasma levels of NLF-1.

**Table 3:** Plasma levels of NLF-1 (pg/ml) in ASD subgroups.

Behavioral test	Mild to moderate (mean ± SD)	Severe (mean ± SD)	*p* value
SRS	954.2 ± 213	590.7 ± 304	0.004**
CARS	708.1 ± 363	606.6 ± 308	0.883
SSP	665.1 ± 346	646.4 ± 307	0.373

*SRS* Social Responsiveness Scale, *CARS* Childhood Autism Rating Score, *SSP* Short Sensory Profile. **Correlation is significant at the 0.01 level.

To measure the severity of ASD behaviors, the ASD children were subcategorized into mild to moderate and severe groups according to their SRS scores. The plasma level of NLF-1 in the mild to moderate ASD group was (954.2 ± 213), and NLF-1 in the severe ASD group was (590.7 ± 304). Mann–Whitney analysis showed that plasma levels of NLF-1 were significantly higher in the mild to moderate ASD group than in the severe ASD group (*p* < 0.05).

ASD children were also categorized according to CARS score, which measures the severity of the disease, into mild to moderate and severe subgroups. Children in the moderate group had plasma NLF-1 levels of (708.1 ± 363), while children with severe ASD had NLF-1 levels of (606.6 ± 308). The correlation test showed that the plasma levels of NLF-1 were higher in children with moderate severity than children with severe ASD, but this correlation did not reach significance.

Sensory impairment in ASD children was measured by SSP and correlated with plasma levels of NLF-1. ASD children were subcategorized according to their performance in SSP into ASD children with typical sensory performance (normal) and ASD children with abnormal sensory performance. Plasma levels of NLF-1 were higher in ASD children with normal sensory performance than in ASD children with abnormal sensory performance, but this correlation was not significant (Table 3).

## Discussion

Biomarkers of neural dysfunction have been extensively studied in ASD as a disease and severity indicator^15^. Channelopathy, such as dysfunctional potassium, sodium and calcium channels, was reported in ASD and was found to be associated with ASD symptoms^3^. For example, a defect in the CN2A gene, which encodes a voltage channel, was reported in ASD and was associated with intellectual disability in ASD children^16,17^.

Another channel associated with neurodevelopmental and psychiatric disorders is the sodium leakage channel (NCA). NCA is mainly expressed in the central nervous system (CNS) and is essential for maintaining normal neuronal excitability. The function of the NCA channel is affected by NLF-1. Abnormal NLF-1 leads to reduced NCA leakage currents and membrane potential hyperpolarization, which affects nerve excitability^4^. Additionally, NCA is physiologically essential for normal neuron excitability in the case of calcium concentration defects, which might lead to epilepsy^18,19^. Other physiological functions that are affected due to dysfunctional NCA are pain sensitivity, intellectual functions, and circadian rhythm, which are also reported in ASD patients^5,8,9,20–25^. In ASD, dysfunction in calcium channels has been extensively reported, which might be linked to reduced NCA activity due to low NLF-1^23^.

This study demonstrates that NLF-1 level is significantly lower in ASD children than in neurotypical children. In an attempt to relate our finding to the severity of ASD, it is interesting to associate the recorded lower NLF-1 to glutamate excitotoxicity as ascertained etiological mechanism. Glutamate clearance from the extracellular space is determined by external glutamate levels and sodium concentrations (1Glu− and 3Na+), usually transported from the synaptic cleft and entering the astrocytic compartment. Because glutamate is removed from the synaptic cleft compartment by sodium-dependent excitatory amino-acid transporters, lower NLF-1, as a regulator of NCA, could disrupt glutamate clearance, overstimulate glutamate receptors, and result in neuronal excitotoxic death^26,27^. Additionally, since NCA-mediated currents at the cellular level drive spontaneous firing in GABAergic neurons and depolarize the resting membrane potential in hippocampal neurons, lower NLF-1, as a regulator of NCA, may be connected to the excitatory/inhibitory imbalance that is a hallmark of ASD^28–30^.

Children in the current study who had mild to moderate ASD had lower plasma levels of NLF-1, and those who had severe ASD had even much lower levels, demonstrating a significant relationship between NLF-1 and the severity of ASD. Our study further tested this finding by correlation analysis, where an increase in SRS score was significantly related to a decrease in NLF-1 level. The severity of ASD behavior in lower NLF-1 levels agrees with a previous report that linked NCA mutation in children with speech and cognitive impairment, which are also the early behavioral signs of ASD^10,31^.

NLF-1 has a negative age correlation (decreases as people age), and this result is consistent with earlier research showing that ASD patients experience worsening symptoms as they age^28^. In addition, compared to younger ASD patients, older ASD patients have smaller brains, a shrinking hippocampus, and worsening anxiety and short-term memory^29–31^.

In conclusion, our results point to a potential connection between the ASD symptoms experienced by our studied patients and the disturbed NCA activity. Although more studies are required to understand the relationship between NCA and the ASD phenotype fully, we are confident that NCA (as measured by NLF-1) plays a role in ASD, the severity of the condition, and aging. This finding will open a new research venue and be the focus of future studies.

## Data Availability

All data generated or analyzed during this study are included in this published article**.**

## References

[1] Lord C, Brugha TS, Charman T, Cusack J, Dumas G, Frazier T (2020). Autism spectrum disorder. Nat. Rev. Dis. Primers..

[2] Al-Mazidi S, Al-Ayadhi LY (2021). Plasma levels of alpha and gamma synucleins in autism spectrum disorder: An indicator of severity. Med. Princ. Pract..

[3] Schmunk G, Gargus JJ (2013). Channelopathy pathogenesis in autism spectrum disorders. Front. Genet..

[4] Cochet-Bissuel M, Lory P, Monteil A (2014). The sodium leak channel, NALCN, in health and disease. Front. Cell Neurosci..

[5] Xie L, Gao S, Alcaire SM, Aoyagi K, Wang Y, Griffin JK (2013). NLF-1 delivers a sodium leak channel to regulate neuronal excitability and modulate rhythmic locomotion. Neuron.

[6] Taurines R, Schwenck C, Westerwald E, Sachse M, Siniatchkin M, Freitag C (2012). ADHD and autism: Differential diagnosis or overlapping traits? A selective review. Atten. Defic. Hyperact. Disord..

[7] Gegelashvili M (2019). Autism and depression (review). Georgian Med. News.

[8] Gao S, Xie L, Kawano T, Po MD, Pirri JK, Guan S (2015). The NCA sodium leak channel is required for persistent motor circuit activity that sustains locomotion. Nat. Commun..

[9] Wang Y, Zhang YB, Liu LL, Cui JF, Wang J, Shum DH (2017). A meta-analysis of working memory impairments in autism spectrum disorders. Neuropsychol. Rev..

[10] Al-Sayed MD, Al-Zaidan H, Albakheet A, Hakami H, Kenana R, Al-Yafee Y (2013). Mutations in NALCN cause an autosomal-recessive syndrome with severe hypotonia, speech impairment, and cognitive delay. Am. J. Hum. Genet..

[11] Mody M, Belliveau JW (2013). Speech and language impairments in autism: Insights from behavior and neuroimaging. N. Am. J. Med. Sci. (Boston)..

[12] Moulton E, Bradbury K, Barton M, Fein D (2019). Factor analysis of the childhood autism rating scale in a sample of two year olds with an autism spectrum disorder. J. Autism Dev. Disord..

[13] Schanding GT, Nowell KP, Goin-Kochel RP (2012). Utility of the social communication questionnaire-current and social responsiveness scale as teacher-report screening tools for autism spectrum disorders. J. Autism Dev. Disord..

[14] Williams ZJ, Failla MD, Gotham KO, Woynaroski TG, Cascio C (2018). Psychometric evaluation of the short sensory profile in youth with autism spectrum disorder. J. Autism Dev. Disord..

[15] Eissa N, Sadeq A, Sasse A, Sadek B (2020). Role of neuroinflammation in autism spectrum disorder and the emergence of brain histaminergic system. Lessons also for BPSD?. Front. Pharmacol..

[16] Spratt PWE, Alexander RPD, Ben-Shalom R, Sahagun A, Kyoung H, Keeshen CM (2021). Paradoxical hyperexcitability from NaV1.2 sodium channel loss in neocortical pyramidal cells. Cell Rep..

[17] Li J, Cai T, Jiang Y, Chen H, He X, Chen C (2016). Genes with de novo mutations are shared by four neuropsychiatric disorders discovered from NPdenovo database. Mol. Psychiatry..

[18] Chua HC, Wulf M, Weidling C, Rasmussen LP, Pless SA (2020). The NALCN channel complex is voltage sensitive and directly modulated by extracellular calcium. Sci. Adv..

[19] Cang, C., B. Lu, and D. Ren, Epilepsy-associated mutations in the calcium-sensing receptor disrupt the regulation of NALCN sodium-leak channel by extracellular calcium in neurons*. bioRxiv*. 2020.11.07.372623 (2020).

[20] Pinggera A, Mackenroth L, Rump A, Schallner J, Beleggia F, Wollnik B (2017). New gain-of-function mutation shows CACNA1D as recurrently mutated gene in autism spectrum disorders and epilepsy. Hum. Mol. Genet..

[21] Clarke C (2015). Autism spectrum disorder and amplified pain. Case Rep. Psychiatry..

[22] Pinato L, Galina Spilla CS, Markus RP, da Silveira Cruz-Machado S (2019). Dysregulation of circadian rhythms in autism spectrum disorders. Curr. Pharm. Des..

[23] Liao X, Li Y (2020). Genetic associations between voltage-gated calcium channels and autism spectrum disorder: A systematic review. Mol. Brain.

[24] Lozic B, Johansson S, Lovric Kojundzic S, Markic J, Knappskog PM, Hahn AF (2016). Novel NALCN variant: Altered respiratory and circadian rhythm, anesthetic sensitivity. Ann. Clin. Transl. Neurol..

[25] Li J, Chen Y, Liu J, Zhang D, Liang P, Lu P (2021). Elevated expression and activity of sodium leak channel contributes to neuronal sensitization of inflammatory pain in rats. Front. Mol. Neurosci..

[26] Flanagan B, McDaid L, Wade J, Wong-Lin K, Harkin J (2018). A computational study of astrocytic glutamate influence on post-synaptic neuronal excitability. PLoS Comput. Biol..

[27] De Pittà M, Brunel N (2016). Modulation of synaptic plasticity by glutamatergic gliotransmission: A modeling study. Neural Plast..

[28] El-Ansary A, Al-Ayadhi L (2014). GABAergic/glutamatergic imbalance relative to excessive neuroinflammation in autism spectrum disorders. J. Neuroinflamm..

[29] Montanari M, Martella G, Bonsi P, Meringolo M (2022). Autism spectrum disorder: Focus on glutamatergic neurotransmission. Int. J. Mol. Sci..

[30] Lu B, Su Y, Das S, Liu J, Xia J, Ren D (2007). The neuronal channel NALCN contributes resting sodium permeability and is required for normal respiratory rhythm. Cell.

[31] Böhm J, Diefenbacher A, Heinrich M, Sappok T (2019). Autism spectrum disorders in adults with intellectual disabilities: Frequencies and characteristics. Nervenarzt..

